# The ecological adaptation of the unparalleled plastome character evolution in slipper orchids

**DOI:** 10.3389/fpls.2022.1075098

**Published:** 2022-12-20

**Authors:** Chao Hu, Zhenbin Jiao, Xinyan Deng, Xiongde Tu, Aixian Lu, Chengzhi Xie, Kai Jiang, Xinhua Zeng, Zhong-Jian Liu, Weichang Huang, Yibo Luo

**Affiliations:** ^1^ State Key Laboratory of Systematic and Evolutionary Botany, Institute of Botany, Chinese Academy of Sciences, Beijing, China; ^2^ Eastern China Conservation Centre for Wild Endangered Plant Resources, Shanghai Chenshan Botanical Garden, Shanghai, China; ^3^ University of Chinese Academy of Sciences, Beijing, China; ^4^ College of Forestry, Fujian Agriculture and Forestry University, Fuzhou, China; ^5^ Key Laboratory of Orchid Conservation and Utilization of National Forestry and Grassland Administration at College of Landscape Architecture and Art, Fujian Agriculture and Forestry University, Fuzhou, China; ^6^ China National Botanical Garden, Beijing, China

**Keywords:** cypripedioideae, photosynthetic plant, climate change, adaptation, NDH complex, gene loss, pseudogenization

## Abstract

Plastomes may have undergone adaptive evolution in the process of plant adaptation to diverse environments, whereby species may differ in plastome characters. Cypripedioideae successfully colonized distinct environments and could be an ideal group for studying the interspecific variation and adaptive evolution of plastomes. Comparative study of plastomes, ancestral state reconstruction, phylogenetic-based analysis, ecological niche modelling, and selective pressure analysis were conducted to reveal the evolutionary patterns of plastomes in Cypripedioideae and their relationship with environmental factors. The plastomes of the three evolved genera had reduced plastome size, increased GC content, and compacted gene content compared to the basal group. Variations in plastome size and GC content are proved to have clear relationships with climate regions. Furthermore, ecological niche modelling revealed that temperature and water factors are important climatic factors contributing to the distributional difference which is directly correlated with the climate regions. The temperature-sensitive genes *ndh* genes, *infA*, and *rpl20* were found to be either lost/pseudogenized or under positive selection in the evolved groups. Unparalleled plastome character variations were discovered in slipper orchids. Our study indicates that variations in plastome characters have adaptive consequences and that temperature and water factors are important climatic factors that affect plastome evolution. This research highlights the expectation that plants can facilitate adaptation to different environmental conditions with the changes in plastome and has added critical insight for understanding the process of plastome evolution in plants.

## Introduction

Genomic characters such as genomic size, GC content, and gene content exhibit pronounced variation among angiosperms (Šmarda et al., 2008; Travnicek et al., 2019). Genome size variation has been correlated with some eco-physiological characters such as cell size, duration of cell division, seed mass, leaf persistence, and growth rate (Morgan and Westoby, 2005; Beaulieu et al., 2007; Beaulieu et al., 2008; Knight et al., 2010; Šímová and Herben, 2012; Tenaillon et al., 2016; Travnicek et al., 2019). These eco-physiological characters are considered the combined result of ecological, physiological, and morphological selection processes at the molecular level (Knight et al., 2005). Moreover, they are probably involved in ecological and evolutionary processes, such as adaptive evolution, speciation, and diversification (Morgan and Westoby, 2005; Suda et al., 2015; Bilinski et al., 2018; Pellicer et al., 2018; Simonin and Roddy, 2018; Travnicek et al., 2019). GC content may also affect gene functioning and species ecology (Šmarda et al., 2014). GC-rich species are favoured in extreme climates, such as cold and dry climates, as well as in environments that show strong temperature fluctuations (Šmarda et al., 2014; Veleba et al., 2017). Functional gene content can be increased by duplication events such as whole-genome duplication or polyploidization (Panchy et al., 2016) or arise *de novo* from intergenic space (Heinen et al., 2009) or under other mechanisms (True and Carroll, 2002; Wray et al., 2003), and gene content can also decrease when involved in loss or pseudogenization events. Gene content variations, regardless of duplication or degradation, are suggested to have effects on the adaptive evolution process in plants (Wang et al., 2019; Helsen et al., 2020; Bohutinska et al., 2021; Li et al., 2022).

The plastome is an important part of the plant genome that is involved in photosynthesis and is also an ideal resource for plant evolutionary-biology studies concerning its specific features such as uniparental inheritance and low evolutionary rate (Wolfe et al., 1987; Drouin et al., 2008; Smith, 2015; Gitzendanner et al., 2018). In most photosynthetic land plants, plastomes range from 140 to 160 kb, containing approximately 113 genes, and are typically partitioned into four regions, including a large single copy, a small single copy, and two inverted repeats (Kim et al., 2020). The plastome is thought to be conserved in size, structure, gene content, and linear order of genes (Wicke et al., 2011; Li et al., 2019). However, plastome characters are found to be different among many species’ lineages (Labiak and Karol, 2017; Kim et al., 2020). Environmental factors or resources such as light, temperature, water, and nutrition can also affect the photosynthetic process (Mayoral et al., 2015; Singh et al., 2018) and play an important role in plastome evolution (Wu et al., 2009; Hu et al., 2015; Ivanova et al., 2017) or even drive speciation (Zupok et al., 2021). Thus, plastome characters, such as plastome size, GC content, and even functional gene content may differ among species/lineages (Labiak and Karol, 2017; Kim et al., 2020) and can provide valuable information that elucidates the patterns of genetic variation in spatiotemporal evolution of land plants (Wu et al., 2012; Hu et al., 2015). However, to date, the evolutionary pattern of plastome character variation and its association with adaptive evolution have rarely been studied compared with those of the nuclear genome.

Slipper orchids (Cypripedioideae, Orchidaceae) are probably the best-characterized orchid subfamily (Cox et al., 1998), including five genera with approximately 200 species that have successfully colonized diverse habitats throughout the tropical and temperate northern hemisphere (Pridgeon et al., 1999). Within Cypripedioideae, *Cypripedium* species occur in the temperate zones of Eurasia and North America, mainly in the alpine area. *Paphiopedilum* extends in tropical and subtropical zones of the karst area from India eastwards to southern China and throughout southeast Asia (Pridgeon et al., 1999). *Mexipedium*, *Phragmipedium*, and *Selenipedium* are distributed in tropical zones of South and North America. They have experienced great environmental changes since their divergence at approximately 65 Mya (Kim et al., 2020). Lineage diversification of Cypripedioideae was found in the Oligocene (Kim et al., 2020) in a period of great temperature variation (Zachos et al., 2001). Thus, climate change has been suggested to contribute to the adaptive evolution of slipper orchids (Guo et al., 2015; Liu et al., 2021). The appropriate species number, long evolutionary history, and widespread distribution make Cypripedioideae an ideal group for adaptive evolutionary studies. The plastomes of Cypripedioideae have been investigated in many studies, and plastome character variations have been confirmed in this group (Kim et al., 2015b; Vu et al., 2020; Guo et al., 2021a; Guo et al., 2021b; Liu et al., 2022). However, due to a lack of comparison at the large-scale subfamily level and the association with climatic factors, the evolutionary patterns of plastome characters in slipper orchids and their potential implications for environmental adaptation remain unclear.

In this study, we sampled 113 taxa in Cypripedioideae and conducted plastome assembling, plastome character comparison, ancestral state reconstruction, phylogeny-based regression and ecological niche modelling to address the following questions: 1) What are the evolutionary patterns of plastome size, GC content, and other characters in Cypripedioideae? 2) Are plastome character variations associated with environmental adaptation?

## Materials and methods

### Taxon sampling, DNA extraction, and sequencing

Samples of 113 taxa from Cypripedioideae (**Table S1**) were sequenced. Leaf samples were collected in a greenhouse and dried on silica gel. Total genomic DNA was extracted from sampled leaves using a plant total genomic DNA kit (Tiangen, Beijing, China) following the manufacturer’s protocol. High-quality DNA samples (OD_260/280_ = 1.8~2.0, OD_260/230_ ≥ 2.0) were used to construct the sequencing library. The libraries were prepared using the TruSeq Nano DNA Library Prep Kit (Illumina, San Diego, CA) following the manufacturer’s protocol. Paired-end reads of 2 × 150 bp were sequenced using the HiSeq 4000 system (Illumina) at Shanghai Major Biopharm Technology Co., Ltd.

Five *Cypripedium* species and one *Paphiopedilum* species, which could not be sampled, were obtained from NCBI and added to the analyses. Finally, the ingroup contained four genera, 110 species, and two natural hybrids; in all, 119 taxa represented 80% of the genera and 56% of the species in Cypripedioideae and 91 taxa were included in *Paphiopedilum* which contained 85 species and a natural hybrid, representing 79% of the species. In turn, there were 18 *Cypripedium* species and one hybrid (21 taxa) representing 33% of the species in the genus. For *Phragmipedium*, six species (six taxa) were sampled representing 25% of the species belonging to this genus. Last, we sampled the only species known of the genus *Mexipedium*. Outgroup species were chosen based on the phylogenetic positions of Orchidaceae inferred by Chase et al. (2015) and Kim et al. (2020). Twelve species from the allied genera in Orchidaceae and five species from other families (**Table S1**) were obtained from NCBI and used for subsequent analyses.

### Plastome assembly, annotation, and plastome character detection

A *de novo* assembly for each species was performed using GetOrganelle (Jin et al., 2020) and checked manually using Bandage (Wick et al., 2015). Plastomes were annotated using DOGMA (Wyman et al., 2004) with a subsequent manual correction for start or stop codons and intron or exon boundaries in Geneious (Kearse et al., 2012) with *Paphiopedilum purpuratum* (MN535015) and *Cypripedium calceolus* (MN602053) as references. Finally, tRNA genes were confirmed using tRNAscan-SE 1.21 (Schattner et al., 2005). The stop codon in each gene was checked to confirm whether pseudogenization had occurred in different species.

The statistics of the whole plastome size and sizes of large single copy (LSC) region, small single copy (SSC) region, inverted repeat (IR) region as well as their GC content were conducted in Geneious v2020.2.4 (Biomatters, Inc., Auckland, New Zealand; http://www.geneious.com). We used the Perl script MISA (https://webblast.ipk-gatersleben.de/misa/) (Thiel, 2003) to detect the SSR numbers. The thresholds for mono-, di-, tri-, tetra-, pent- and hexanucleotide SSRs were set to 10, 5, 4, 3, 3, and 3 repeat units, respectively (Dong et al., 2018). As the assemblies of *Paphiopedilum sugiyamanum*, *P. glaucophyllum*, and *P. primulinum* were not successful in some intergenetic regions, we excluded them from these analyses.

### Ancestral state reconstruction of plastome characters

Considering the high variability in the intergenic regions, 79 protein-coding genes were extracted by PhyloSutie v1.1.16 (Zhang et al., 2020) and used for further phylogenetic analyses. The alignment was performed using MAFFT v7.455 (Katoh and Standley, 2014) with default parameters. The best-fit nucleotide substitution model TVM+I+G was estimated using jModelTest 2.1.10 (Posada, 2008) under the corrected Akaike information criterion (AIC). Then, the best-fit model was used for the subsequent analysis. Phylogenetic reconstruction was conducted using RAXML v7.0.4 (Stamatakis, 2006) with 1000 bootstrap replicates.

Ancestral state reconstruction for plastome size, GC content (both for total plastome and three different parts), and number of SSRs were conducted by the “fastAnc” function and visualized on the phylogeny tree with the “contMap” function in the R package phytools v0.7-90 (Revell, 2013). *Paphiopedilum sugiyamanum*, *P. glaucophyllum*, and *P. primulinum* were excluded from this and subsequent analyses.

### Phylogenetic signals in plastome characters

The Pagel *λ*-coefficient was calculated by the “fitContinuous” function in the R package geiger (Harmon et al., 2008) for plastome characters. The value of *λ* varies from 0 to 1, such that the higher the *λ* value, the greater the correlation between phylogeny and characters.

### Relationship between plastome characters and ecological variables

The regression relationships between plastome characters and ecological variables were tested by performing the phylogenetic generalized least squares procedure (PGLS) using the “pgls” function in the R package caper (Orme et al., 2013). Four ecological variables were analysed to check whether they contributed to the variation in plastome size and GC content. Climate regions 'tropical', 'subtropical' and 'temperate' for plants growing exclusively or predominantly in respective areas. Distribution countries are from WSCP (https://wcsp.science.kew.org) and the maximum and minimum altitudes are from the literature.

Occurrence records of Cypripedioideae were obtained from the Global Biodiversity Information Facility (GBIF; https://www.gbif.org/zh/). Duplicates, outliers, zero coordinates, and erroneous occurrences in the sea were obtained using the function “CoordinateCleaner” in R (Zizka et al., 2019). Then, the data were manually adjusted to ensure at most one valid point in each grid cell. The total numbers of records are 232 and 364 for *Cypripedium* and four other genera of Cypripedioideae, respectively. Minimum, mean, and maximum temperature, precipitation, solar radiation, wind speed, water vapour pressure, and 19 bioclimatic, as well as elevation variables, were downloaded from WorldClim version 2.1 (Fick and Hijmans, 2017) (http://www.worldclim.org/) at a spatial resolution of 10 mins. We then performed an additional Spearman’s correlation analysis to determine if there was a correlation among all variables, and only the highest percent contribution variable was kept when the correlation occurred. MaxEnt version 3.4.4 (Phillips et al., 2017) was used for ecological niche modelling, and model parameters were selected following processes outlined in Wani et al. (2021). ArcMap 10.2 was used to show the potential distribution.

### Positive selection analyses

We used the branch site model of PAML (Yang, 2007) to identify positively selected plastid protein-coding genes. The three evolved genera were defined as the foreground branch and *Cypripedium* was defined as the background branch. Two branch site models i.e., an alternative model and a null model were tested with the branch site specific ω varying freely or fixed at 1. Based on a Bayes empirical Bayes (BEB) result with a posterior probability greater than 0.95, the LRT values were calculated to test whether the likelihood of the alternative model was significantly different from that of the null model. The *p* values were calculated using a chi-square distribution with one degree of freedom. Genes with *p* < 0.05 were selected as candidate positives.

## Results

### Variation in plastome characters within Cypripedioideae

**Figure 1:**
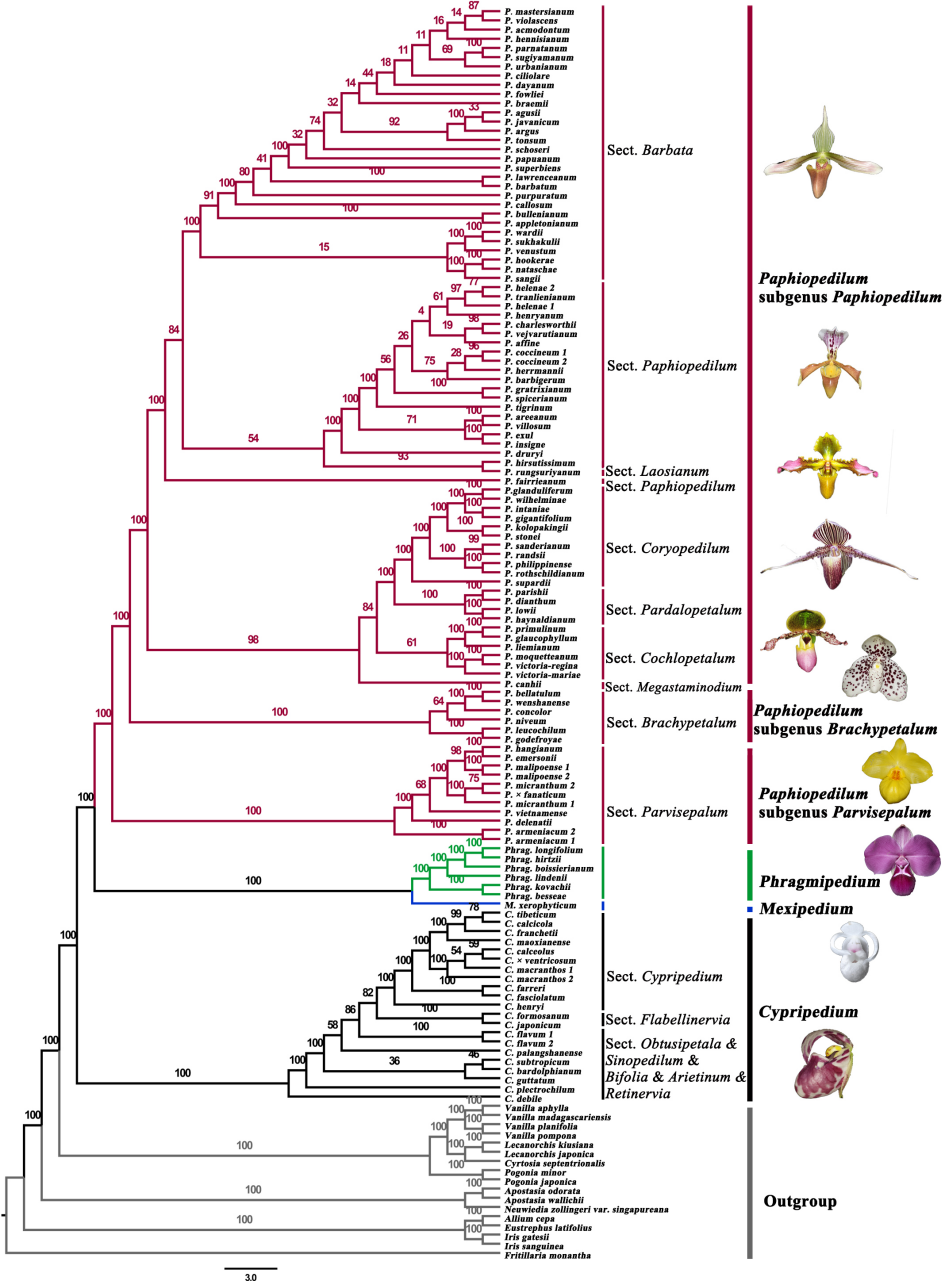
Maximum likelihood tree of Cypripedioideae based on the protein-coding genes in plastomes.

**Figure 2:**
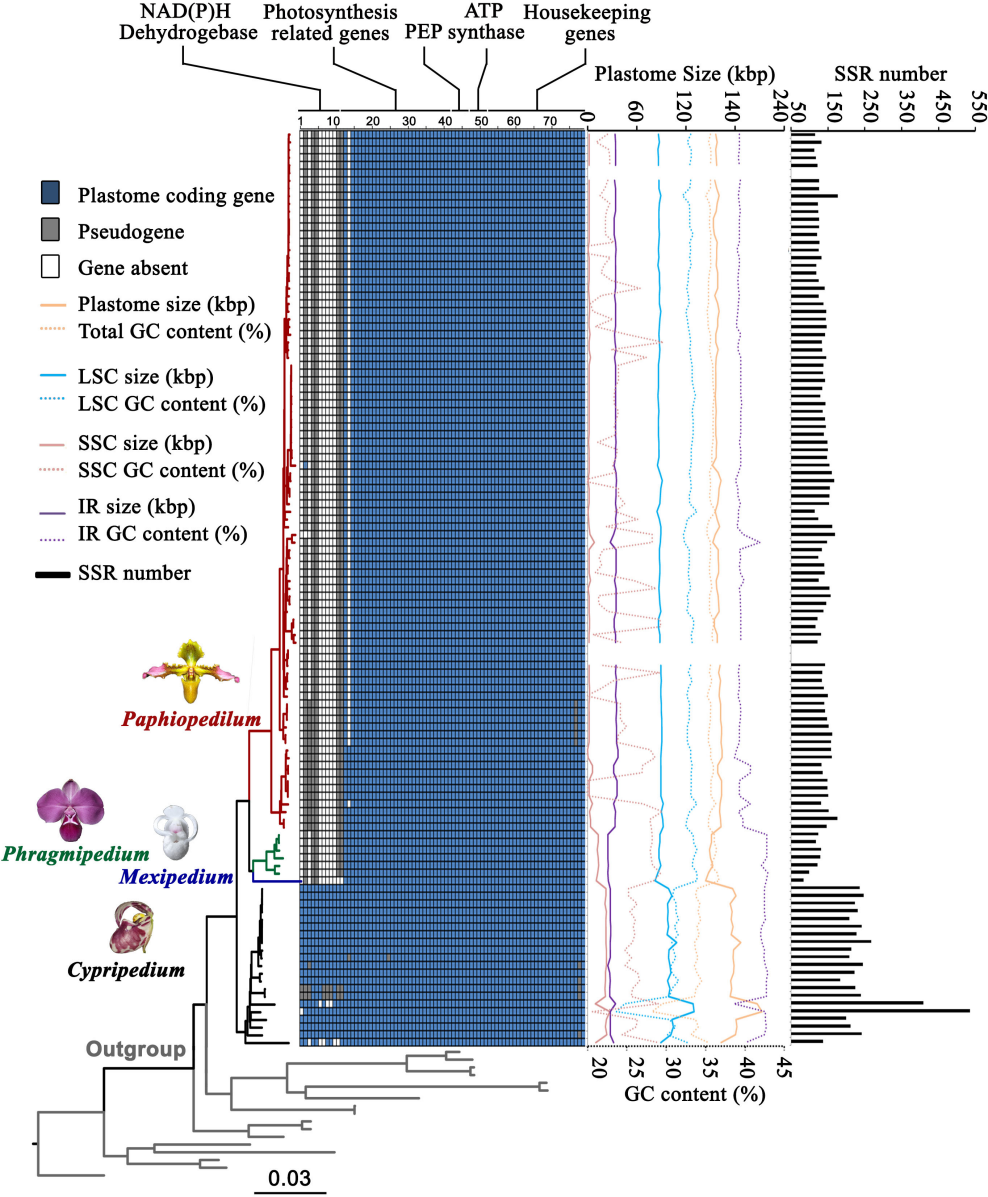
Gene content, plastome size, GC content, and SSR number variation of plastomes in Cypripedioideae. Number 1-79 represent 1 *ndhA*, 2 *ndhB*, 3 *ndhC*, 4 *ndhD*, 5 *ndhE*, 6 *ndhF*, 7 *ndhG*, 8 *ndhH*, 9 *ndhI*, 10 *ndhJ*, 11 *ndhK*, 12 *ccsA*, 13 *cemA*, 14 *petA*, 15 *petB*, 16 *petD*, 17 *petG*, 18 *petL*, 19 *petN*, 20 *psaA*, 21 *psaB*, 22 *psaC*, 23 *psaI*, 24 *psaJ*, 25 *psbA*, 26 *psbB*, 27 *psbC*, 28 *psbD*, 29 *psbE*, 30 *psbF*, 31 *psbH*, 32 *psbI*, 33 *psbJ*, 34 *psbK*, 35 *psbL*, 36 *psbM*, 37 *psbN*, 38 *psbT*, 39 *psbZ*, 40 *rbcL*, 41 *ycf3*, 42 *ycf4*, 43 *rpoA*, 44 *rpoB*, 45 *rpoC1*, 46 *rpoC2*, 47 *atpA*, 48 *atpB*, 49 *atpE*, 50 *atpF*, 51 *atpH*, 52 *atpI*, 53 *rpl2*, 54 *rpl14*, 55 *rpl16*, 56 *rpl20*, 57 *rpl22*, 58 *rpl23*, 59 *rpl32*, 60 *rpl33*, 61 *rpl36*, 62 *rps2*, 63 *rps3*, 64 *rps4*, 65 *rps7*, 66 *rps8*, 67 *rps11*, 68 *rps12*, 69 *rps14*, 70 *rps15*, 71 r*ps16*, 72 *rps18*, 73 *rps1*9, 74 *accD*, 75 *clpP*, 76 *infA*, 77 *matK*, 78 *ycf1* and 79 *ycf2*. The dark blue squares represent existing coding genes, the grey squares represent pseudogenes and the white squares represent absent genes.

We recovered complete plastomes for the Cypripedioideae. The plastomes of three evolved genera (*Paphiopedilum*, *Phragmipedium*, and *Mexipedium*) are reduced in size compared to *Cypripedium* (**Figures 1**, **2**; **Table S1**), ranging from 144,335 to 212,668 bp. The average plastome size of the four genera showed the following trend: *Cypripedium* (180,352 bp) > *Paphiopedilum* (158,659 bp) > *Phragmipedium* (150,213 bp) > *Mexipedium* (144,335 bp). In general, the complete plastome of most species has a single circular molecule with a quadripartite structure consisting of a large single copy (LSC) region, a small single copy (SSC) region, and two inverted repeat (IR) regions. The sizes of the LSC, SSC, and IR regions within Cypripedioideae (**Table S1**) were 82,348-129,998, 336-27,414, and 24,517-37,043 bp, respectively. Plastomes of *Paphiopedilum* show clear IR expansion and SSC contraction (**Figure 2**).

The plastid gene content in the subfamily Cypripedioideae varied among genera in the subfamily. The plastome of Cypripedioideae contained 134 genes, 26 of which were duplicated in the IR regions. The unique genes included 30 distinct tRNA genes, four ribosomal RNA (rRNA) genes, and 79 protein-coding genes. Gene loss and pseudogenization were common in Cypripedioideae (**Figure 2**). Eleven *ndh* genes, as well as *cemA*, *psaJ*, *infA*, and *matK* were lost or turned into pseudogenes in different genera or species. In *Cypripedium*, 11 *ndh* genes were retained, but some of them lost function in four species. Only five *ndh* genes currently exist in *Paphiopedilum*, and they lost function in most species, while the *infA* gene in subgenus *Brachypetalum* is a pseudogene, and *cemA* is absent from all species of subgenus *Paphiopedilum*. *matK* turned into a pseudogene in six *Paphiopedilum* species. All *ndh* genes are absent or have become pseudogenes in the genus *Phragmipedium*, while two *ndh* genes (*ndhB* and *ndhD*) are now both pseudogenes in the genus *Mexipedium*.

Regarding GC content, we observed significant differences among Cypripedioideae genera. The GC content of the whole plastome ranged from 28.2% to 36.7%. The evolutionary trend of the average GC content was *Cypripedium* (33.5%) < *Paphiopedilum* (35.3%) < *Phragmipedium* (36.2%) < *Mexipedium* (36.7%). Furthermore, the GC contents in the LSC, SSC, and IR regions were 23.7%-33.9%, 16.9%-42.1% and 38.6%-42.9%, respectively (**Figure 2**; **Table S1**). In addition, the total number of SSR loci varied among Cypripedioideae genera. We identified 18,239 SSR loci consisting of 38.5% mono-SSRs, 20.3% di-SSRs, 16.3% tri-SSRs, 13.8% tetra-SSRs, 6.9% penta-SSRs, and 4.3% hexa-SSRs (**Figure 2**; **Table S1**). The genus *Cypripedium* showed the largest number of repeats, followed by *Paphiopedilum*, *Phragmipedium*, and *Mexipedium*. In particular, *Cypripedium subtropicum* and *Mexipedium xerophyticum* showed the highest and lowest numbers of repeats, respectively. A large number of AT-biased repeat sequences were found in *Cypripedium* which contributed to the high SSR numbers in this lineage.

Combining these results, we found that evolutionary patterns of plastome characters in the basal group are different from those in the evolved groups in Cypripedioideae.

### The ancestral states of plastome characters

Phylogenetic analysis of 79 protein-coding genes yielded a robust tree for the four genera of Cypripedioideae (**Figures 1**, **2**; **Table S1**). The results showed that the four genera of Cypripedioideae in this study were monophyletic. *Cypripedium* was a basal group of the other three genera. The clade including *Phragmipedium* and *Mexipedium* was supported by high bootstrap (ML_BS_=100%), which showed a close relationship between the two genera. In turn, *Paphiopedilum* formed a sister group of *Phragmipedium* and *Mexipedium*, and three subgenera of *Paphiopedilum* clustered closely together (ML_BS_=100%).

**Figure 3:**
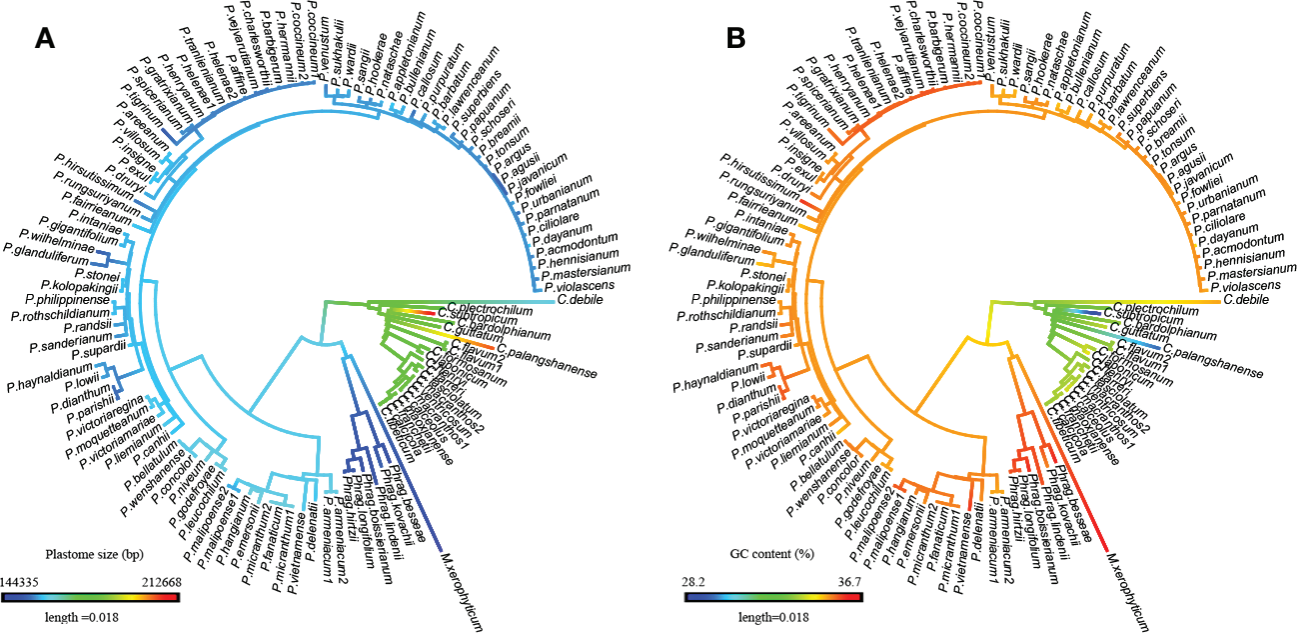
Evolutionary patterns of plastome size and GC content in Cypripedioideae as inferred from ancestral state reconstruction. **(A)** Plastome size, **(B)** GC content.

Based on ancestral state reconstruction analysis, the evolution of plastome size, GC content, and SSR number followed different trends across the four genera in Cypripedioideae (**Figures 3**; **S1**). Plastome contraction and expansion are apparent in different lineages compared to the estimated ancestral state of a medium-sized plastome (170,541 bp) for Cypripedioideae. Apparent plastome expansion was observed in the basal group, *Cypripedium*, while the opposite trend of genome downsizing occurred in three evolved lineages, especially in *Phragmipedium* and *Mexipedium*. The evolution of GC content also acts specifically in the lineage. The trend of GC content evolution either decreased in *Cypripedium* or increased in *Paphiopedilum*, *Phragmipedium*, and *Mexipedium*. The ancestral states of other plastome characters are shown in **Figure S1**. Most of these characters showed variation in different clades compared to ancestral states. However, the evolution of SSC GC content was relatively nonobvious across ancestral states and existing species.

### Phylogenetic signals in plastome characters

To test the correlation between phylogeny and characters, the separate estimate *λ* values of plastome size, LSC size, GC content and SSR number were calculated and are shown in **Table 1**. The estimated *λ* for these characteristics approaches 1 and the *p* values for plastome size and GC content of the total plastome and LSC as well as SSR numbers are under 0.01. These results indicate that plastome size, GC content, and SSR number showed strong phylogenetic signals. Conversely, phylogenetic signals of the size and GC content of the SSC and IR regions were not significant.

**Table 1:** Phylogenetic signals of plastome characters.

Plastome characters	Lambda (*λ*)	*p* value
Plastome size	0.974	7.17E-29**
LSC size	0.964	7.18E-37**
SSC size	1	1.00E+00
IR size	1	1.00E+00
Total GC content	0.994	1.01E-14**
LSC GC content	0.994	7.61E-24**
SSC GC content	1	1.00E+00
IR GC content	1	1.00E+00
Total SSR	0.992	1.49E-07**

'**' indicate p value less than 0.01.

### Relationship between plastome characters and ecological variables

A significant portion of plastome size variation could be explained by the climate region of species in Cypripedioideae [*F* statistic = 5.35, *p* < 0.001, Lambda = 0.967 (0.941-0.981)] (**Table 2**), which explained 9.754% of the variation. The climate region is also related to the GC content variation and the explained proportion is 7.475%. Considering that climate regions are classified into tropical, subtropical, and temperate, and the temperature and precipitation are the main climate factors used to divide those three different climate regions. Thus climatic factors such as temperature and water factors may be associated with the variation in these two plastome characters. However, the factors of distribution and altitude were not related to the two plastome characters. These results indicated that plastome characters can be affected by environmental factors such as temperature, but they could not simply be interpreted by the factors of distribution and altitude.

**Table 2:** Summary of PGLS regression models explaining variation in plastome size and GC content.

	Explanatory variable	*F* statistic	*p* value	Lambda	Explained variation (%)
Plastome size	Climate region	5.350	0.006**	0.967 (0.941, 0.981)	9.754
	Distribution	1.111	0.357	0.969 (0.947, 0.982)	18.360
	Maximum altitude	1.909	0.170	0.971 (0.949, 0.984)	1.874
	Minimum altitude	0.698	0.878	0.972 (0.950, 0.985)	27.880
GC content	Climate region	3.999	0.021*	0.992 (0.984, 0.996)	7.475
	Distribution	1.242	0.252	0.992 (0.984, 0.996)	20.09
	Maximum altitude	0.040	0.843	0.993 (0.986, 0.997)	0.0395
	Minimum altitude	0.999	0.490	0.996 (0.990, 0.998)	35.630

'*' and '**' indicate p value less than 0.05 and 0.01, respectively.

**Figure 4:**
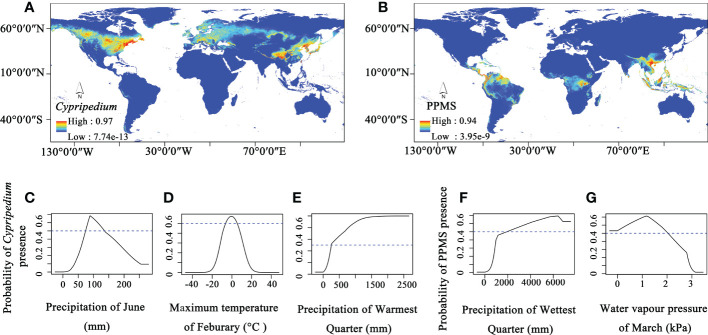
The ecological niche modelling of Cypripedioideae. **(A)** Potential distribution area of *Cypripedium*, **(B)** Potential distribution area of *Paphiopedilum*, *Phragmipedium*, *Mexipedium*, and *Selenipedium* (PPMS), **(C–E)** Three major climate factors contribute to the distribution of *Cypripedium*, **(F, G)** and two major climate factors contribute to the distribution of PPMS.

Therefore, we further conducted ecological niche modelling to investigate environmental factors that affect the distributions and niches of *Cypripedium* and the other four evolved genera. The modelling results show high AUC values for both *Cypripedium* (0.960) and the other four genera of Cypripedioideae (0.977), indicating that the model predictions are accurate. The potential distribution area of *Cypripedium* ranges from North America and Eurasia with two distribution hotspot areas (North America and East Asia, **Figure 4A**). Three environmental factors including the precipitation in June, the maximum temperature in February, and the precipitation in the warmest quarter contribute 36%, 34.1% and 11.9% of the distribution of *Cypripedium*, respectively (**Figures 4C–E**). When the precipitation in June ranges from 74.3 to 136.1 mm, the maximum temperature in February changes between -6.4°C and 5.2°C and the precipitation in the warmest quarter is higher than 257.3 mm, the probability presence of *Cypripedium* is higher than 0.5. Therefore, the distribution of *Cypripedium* is related to temperature and precipitation.

The optimum distribution area of the other four genera included not only Central and South America and Southeast Asia but also Central Africa (**Figure 4B**). Precipitation of the wettest quarter and water vapour pressure of March contribute 44.3% and 31.4% of the four genera distributions, respectively (**Figures 4F, G**). When the precipitation of the wettest quarter is higher than 1925.30 mm and the water vapour pressure of March is under 2.03 kPa, the area is potentially suitable for the survival of these four genera.

From the results, we found that the distribution of both *Cypripedium* and the other four genera of Cypripedioideae are affected by water factors but only *Cypripedium* is affected by temperature. Therefore, both water and temperature factors contribute to shaping the current distribution pattern of slipper orchids, and the temperature is an important climatic factor that results in the distributional difference between the basal group and evolved groups in Cypripedioideae.

### The positively selected genes and sites

**Figure 5:**
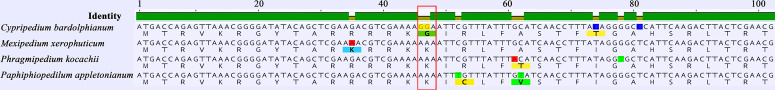
The positively selected site of *rpl20* from representative species in slipper orchids. Each species can represent all the types of the positively selected 16^th^ amino acid (in red frame) in the genus, respectively.

Selective pressure analyses for all protein-coding genes (except 11 *ndh* genes) were performed. The *p* values for *infA* and *rpl20* are under 0.05 which demonstrates that the two genes are positively selected in the foreground branch (**Table 3**). A positively selected site was found in *rpl20*. The 16th amino acid was glycine in *Cypripedium* and lysine in other genera (**Figure 5**).

**Table 3:** Positively selected genes and sites in the plastomes of slipper orchids.

Gene	Alternative model (Ln L/np)	Null model (Ln L/np)	df	*p* value	Positively selected sites
*infA*	-579.797/229	-577.875/228	1	0.050	–
*rpl20*	-1232.410/240	-1235.452/239	1	0.014	16 G 0.962*

'*' indicate p value less than 0.05.

## Discussions

### Distinct evolutionary patterns of plastome characters in Cypripedioideae

The plastome characters of slipper orchids show distinct evolutionary patterns. The plastome size in *Cypripedium* is the largest and in *Mexipedium* is the smallest. While GC content shows the opposite trend, the highest in *Mexipedium* and the lowest in *Cypripedium*. And the ancestral state reconstruction analyses (**Figures 3**; **S1**) showed the evolution patterns in the basal group and the three evolved groups were also distinct. Plastome size expanded in *Cypripedium* but contracted in the three evolved groups. While GC content increased in the three evolved groups and decreased in *Cypripedium*. Gene content is also different between the basal group and the three evolved groups. The most significant one is the loss/pseudogenization in three evolved groups but not in *Cypripedium*.

From the basal group to evolved groups in Cypripedioideae, there is an evolutionary trend of reduced plastome size. Previous studies suggested that plastome size variation in Cypripedioideae can be achieved from the variation in IR regions or noncoding regions (Kim et al., 2020; Wei et al., 2021), gene loss (Kim et al., 2020; Li et al., 2020) and repeat sequences (Dugas et al., 2015; Guo et al., 2021b). In our study, the relatively small plastome size of *Mexipedium* and *Phragmipedium* was caused by gene loss and nonexpansion of the IR region. IR region expansion and gene loss are responsible for plastome size in *Paphiopedilum* (Guo et al., 2021a) and abundant AT-rich repeats and nearly no gene loss are the reasons for the expansion of plastome in *Cypripedium* (Kim et al., 2015b; Kim et al., 2020). Therefore, a combination of SSRs (mostly for AT-rich repeats), and contraction/expansion of IRs and gene content are responsible for the plastome size variation in slipper orchids.

GC content is a key genome feature that is associated with fundamental elements of genome organization, such as gene density, mutation rates, GC-biased gene conversion, and deletion mutations (Eyre-Walker and Hurst, 2001; Niu et al., 2017; Yu et al., 2021). The plastid GC content varies greatly among species from 22.67% to 56.50% (Bellot and Renner, 2015; Zhang et al., 2019). In this study, the lowest GC content was observed in *Cypripedium subtropicum* and the highest was found in *Mexipedium xerophyticum.* In contrast to plastome size, increasing GC content is found from the basal group (*Cypripedium*) to the evolved groups. The high SSR number especially the AT-biased repeat sequences in *Cypripedium* (Guo et al., 2021b) is an important reason for the unparalleled GC content in slipper orchids.

The *ndh* genes are often pseudogenized or entirely lost several times during land plant evolution (Wicke et al., 2011). The loss of *ndh* genes was found in many previous studies on orchids which occurred independently during the splitting of different orchid lineages (Luo et al., 2014; Kim et al., 2015a; Lin et al., 2015). In this study, we found that the loss/pseudogenization of cp-*ndh* genes is widespread in the evolved groups of Cypripedioideae but not in the basal group, *Cypripedium*. As plastid gene loss happens sequentially, starting with the *ndh* genes (Barrett et al., 2014; Wicke et al., 2016; Graham et al., 2017), the plastomes of Cypripedioideae are in the early stages of plastid degradation.

### The ecological adaptation of plastome character variation

Phylogenetic-based regressions clearly show that the climate region is related to the variation in plastome characters (plastome size and GC content). Two environmental factors (related to climate regions) are found to be associated with the distribution of slipper orchids. Adaptation to these environmental factors (especially the temperature factor) and their change during history may be the reason for distinct plastome characters and their different evolutionary patterns in slipper orchids. Two temperature-sensitive genes were positively selected and loss/pseudogenization of *ndh* genes in evolved groups confirmed our induction on the adaptive evolution of plastome in slipper orchids.

Selections have favoured small genome sizes for the sake of growth efficiency or competitiveness (Moran, 2002). The reduced plastome sizes are also hypothesized to be an adaptation to the increased competition with other angiosperms (Gray et al., 1998; Comeron, 2001; Sudianto et al., 2020). Therefore, smaller plastome size may be important to these species, such as *Paphiopedilum*, which grows in the karst areas with low light intensity and barren and arid environments compared with *Cypripedium* which lives in alpine habitats with high light, abundant soil nutrients, and water. Species with high GC content were able to live in seasonally cold and/or dry climates, which may indicate that GC-rich DNA has an advantage during cell freezing and desiccation (Šmarda et al., 2014). The unparalleled GC content in slipper orchids could play a role during adaptation to divergent environments. Therefore, plastome size and GC content variation in slipper orchids are associated with adaptation to different environments.

Our study shows obvious ecological niche differentiation between *Cypripedium* and other evolved genera of slipper orchids. The slipper orchids currently occurring in the tropics (*Mexipedium*, *Phragmipedium*, and *Paphiopedilum*) were separated from the temperate distributed *Cypripedium* in the Oligocene (Kim et al., 2020) after the Eocene/Oligocene boundary (Zachos et al., 2001; Christie et al., 2005; Tripati et al., 2005) as the Earth shifted from extremely warm to cold conditions with an apparent decrease in temperature. Based on our ecological niche modelling results, we reveal that the occurrence of both *Cypripedium* and the other four genera of Cypripedioideae are affected by water factors (precipitation and vapourization) and that *Cypripedium* is affected by both temperature and water factors. Thus, for *Cypripedium*, increased tolerance of adaptation to a cool environment has contributed to its repeated dispersal and radiation in temperate zones (Liu et al., 2021). For other genera, especially *Paphiopedilum*, dispersal, and adaptation to the higher temperature tropical environment have contributed to their survival and radiation.

Moreover, the two genes (*infA* and *rpl20*) were fixed by positive selection between the basal and evolved groups in selective pressure analysis. *InfA* is closely associated with the cold stress response. In particular, after cold shock, *de novo* transcription and translation of *infA* contribute to the transient increase in the IF1/ribosome ratio, which is partially responsible for translational bias consisting of the preferential translation of cold-shocked mRNAs in the cold environment (Giangrossi et al., 2007). The large ribosomal protein *rpl20* is sensitive to temperature change. The transcription rate of *rpl20* decreases rapidly under heat stress in maize (Nakajima and Mulligan, 2001). Different selective pressures of *infA* and *rpl20* (with a positively selected site) represent *Cypripedium* and other slipper orchids that have adapted to different environments with different temperatures. Previous studies on leaf anatomical structures and physiological traits associated with temperature and water factors supported this suggestion (Chang et al., 2011; Guan et al., 2011).

The loss/pseudogenization of *ndh* genes in Cypripedioideae may play important roles in different light and temperature adaptations. Another remarkable variation in the plastome of slipper orchids is the loss/pseudogenization of *ndh* genes in evolved genera which is also found in other studies of Cypripedioideae and other orchids (Luo et al., 2014; Kim et al., 2015a; Lin et al., 2015; Kim et al., 2020). Gene loss through pseudogenization can be achieved by relaxed selection (Go et al., 2005; Wertheim et al., 2015). The NDH complex (encoded by *ndh* genes) forms a supercomplex with PSI which mediates one route of the PSI cyclic electron flow (CEF) (Shikanai, 2007; Yamori et al., 2015). Although NDH-dependent CEF is not as important as another CEF (PGR5/PGRL1-dependent), it is an important way to balance the ATP/NADPH production ratio and/or protect both photosystems from stress (Casano et al., 2001; Shikanai, 2007; Yamori et al., 2015). The photosynthesis rate of the *ndh* gene-defective plants will decrease when light intensity changes rapidly (Barth and Krause, 2002; Martín and Sabater, 2010). In addition, the NDH complex is sensitive to temperature change. Yamori et al. (2011) found that rice *crr6* mutants that do not accumulate the NDH complex showed lower values for photosynthetic parameters at a relatively low temperature (20°C), concomitant with a corresponding reduction in plant biomass. Zhang et al. (2011) found slower photosynthetic induction in *Paphiopedilum armeniacum* than in *Cypripedium flavum*. Chang et al. (2011) found that the leaf physiological traits of *Cypripedium* and *Paphiopedilum* have adapted to alpine and karst areas, respectively. These results supported our hypothesis that the NDH complex of the evolved genera of Cypripedioideae may lose its function under different light and temperature stresses in the tropical areas compared to *Cypripedium* in the temperate areas.

Some studies found that the *ndh* genes transferred from the plastid to mitochondria in *Paphiopedilum armeniacum* and *P. niveum* (Lin et al., 2015). Because merely transferring plastid DNA into the nuclear or mitochondrial genome merely is not sufficient for functional gene transfer, plastid genes must also acquire regulatory elements as well as transit peptides (Martin and Herrmann, 1998). Lin et al. (2017) checked the published genome of four species, and copies of cp-*ndh* genes are not present in any of these orchid genomes. Therefore, the NDH-complex is very likely to lose its function, but there is no direct evidence, and further studies are still needed.

In conclusion, Our large-scale plastome trait comparison in slipper orchids revealed that detected plastome characters have no parallel between the basal group (*Cypripedium*) and evolved groups (*Paphiopedilum*, *Mexipedium*, and *Phragmipedium*). The evolutionary patterns of plastome size and GC content are closely associated with climate regions thus corroborating the suggestion that plastome traits have adaptive consequences. These plastome trait variations might impose constraints on phenotypic and physiological evolution through a low-cost strategy. Moreover, ecological niche modelling revealed that climatic factors (i.e., temperature and water factors) are the main factors that contribute to the distribution of different groups. Genes (i.e., *infA*, *rpl20*, and *ndh* genes) in the evolved groups are under positive selection or undergoing loss/pseudogenization through relaxed selection. These genes can be affected by temperature, and the different selective pressures on these genes might also be the consequence of environmental adaptation. Our study might provide a new vision of plastome evolution and help to better understand the adaptation of plastomes. At the same time, provide the impetus for evolutionary biologists to continue to test for the occurrence and effects or adaptation to environmental change.

## Data availability statement

The data presented in the study are deposited in NCBI, accession number OL741710-OL741711, OL854223, OL875115-OL875146, OM066272-OM066348, OM203183.

## Author contributions

YL, CH, and WH planned and designed the research. WH, CH, ZJ, XT, XD, AL, and CX collected samples, performed experiments, and analyzed data. YL, WH, Z-JL, ZJ, KJ, XZ, and CH wrote the manuscript. All authors contributed to the article and approved the submitted version.

## Supplementary material

Supplementary Table 1 Materials used in this study and characters of plastomes.

Supplementary Figure 1 Ancestral state reconstruction of plastome characters.
