# Supplementary figures and images for: The ecological adaptation of the unparalleled plastome character evolution in slipper orchids
Source: Front Plant Sci. 2022 Dec 20;13:1075098. doi: 10.3389/fpls.2022.1075098 (PMC9808092; doi:10.3389/fpls.2022.1075098)

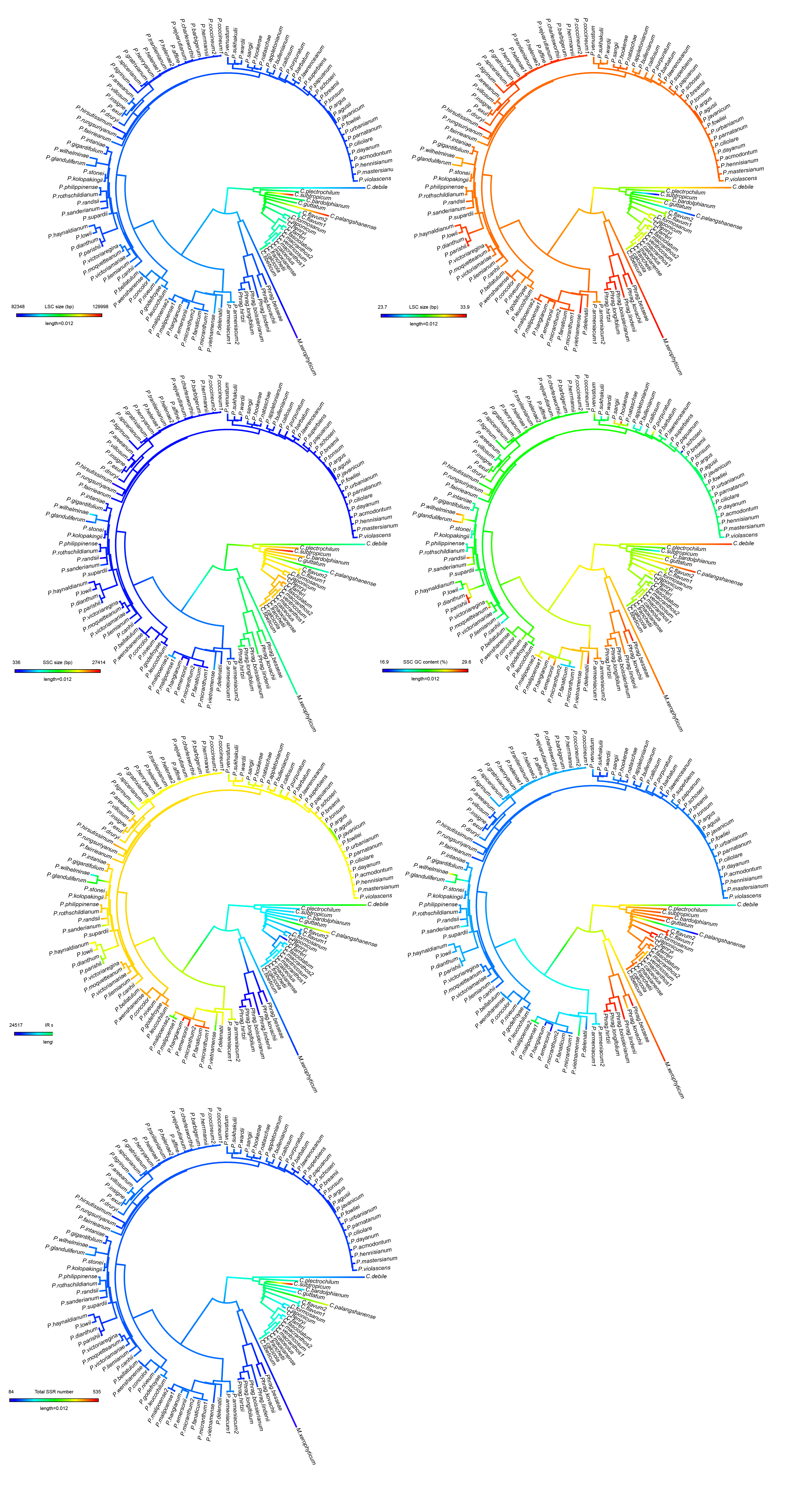

Supplement: Supplementary Figure 1 — Ancestral state reconstruction of plastome characters. [file Image_1.jpeg]
